# The Norfolk Plan

**Published:** 1921-02-26

**Authors:** 


### The Norfolk Plan.

### HOW TO ORGANISE DISTRICT SUPPORT.

The financial difficulty of the Norfolk and Norwich Hospital appears to have stirred the residents of the large agricultural district served by the hospital to a sense of their obligations. For many years, an annual subscription from the squire, a collection in the church on the occasion of the Harvest Thanksgiving Service, the contents of a collecting-box at the local hostelry, and an occasional donation from a grateful patient, were the total support received by the hospital from most of the parishes in Norfolk. Now the small towns and villages are endeavouring to outdo each other in the amount which they can send to the hospital. Whist drives, dances, and entertainments are the foundations upon which most of the villages build for their money-raising efforts, and in the first six weeks of this year the hospital has received £1,650, the result of 102 entertainments of this nature.

### How it Started.

A small town near Norwich for many years had been notorious for the small support given to the hospital in return for the large number of patients annually sent, and on the occasion of a tradesman making a donation upon his son's discharge, the hospital secretary, Mr. Frank Inch, politely informed him that the town in which he resided was on the hospital's "black list," as the cost of the patients treated each year exceeded by £200 the contributions received from the town. The tradesman went away saying he would "alter things!" Within a month he organised a whist drive and a dance which resulted in a cheque for the hospital of nearly £100. The success of this effort awoke a neighbouring town to a sense of its responsibility, and it straightway went "one better." Other towns and villages followed, and now a village of 300 residents is not satisfied until it shall have raised between thirty and forty pounds in a single evening.

### The Use of a Newspaper.

The hospital has a small advertising space at its disposal in the local newspaper every Saturday, and for several weeks now the secretary has endeavoured, by attractive announcements, to bring every parish into "the race." He has called attention to a whist drive held in a room which could not accommodate more than thirty persons, realising £6, to a small parish raising an amount equalling 2s. 5d. per head of the population in one evening, to a cake which sold again and again at a social evening; and in each case he invites parishes to "beat it" if they can. The local newspaper has greatly helped in the campaign, by giving daily prominence to the results of entertainments on behalf of the hospital. An arrangement between the Editor and the hospital secretary ensures that all noteworthy efforts are published in the news columns.

### The Crest of the Wave.

The hospital board of management realises that this present money-raising boom is only a phase, and that sooner or later the "whist-drive fever" (as it has been called) will end, but the hospital authorities are working it for all it is worth while they have the opportunity, and in the meantime are endeavouring to arrange in each parish a systematic weekly collection. The system of one penny per week contributions from employees is being extended to residents in country districts, and already several villages have adopted it. It is found that the interest aroused in the hospital by the holding of an entertainment on its behalf has paved the way for the weekly contribution schemes. The adoption of these in every parish served by the hospital would largely solve the financial difficulties of the present time.

